# Beam focal spot position determination for an Elekta linac with the Agility^®^ head; practical guide with a ready‐to‐go procedure

**DOI:** 10.1002/acm2.12344

**Published:** 2018-05-14

**Authors:** Jacek M. Chojnowski, Lee M. Taylor, Jonathan R. Sykes, David I. Thwaites

**Affiliations:** ^1^ Mid North Coast Cancer Institute Coffs Harbour Health Campus Coffs Harbour NSW Australia; ^2^ Institute of Medical Physics School of Physics University of Sydney Sydney NSW Australia; ^3^ Department of Radiation Oncology Blacktown Cancer & Haematology Centre Blacktown NSW Australia

**Keywords:** beam focal spot, EPID, quality assurance

## Abstract

A novel phantomless, EPID‐based method of measuring the beam focal spot offset of a linear accelerator was proposed and validated for Varian machines. In this method, one set of jaws and the MLC were utilized to form a symmetric field and then a 180^o^ collimator rotation was utilized to determine the radiation isocenter defined by the jaws and the MLC, respectively. The difference between these two isocentres is directly correlated with the beam focal spot offset of the linear accelerator. In the current work, the method has been considered for Elekta linacs. An Elekta linac with the Agility^®^ head does not have two set of jaws, therefore, a modified method is presented making use of one set of diaphragms, the MLC and a full 360^o^ collimator rotation. The modified method has been tested on two Elekta Synergy^®^ linacs with Agility^®^ heads and independently validated. A practical guide with instructions and a MATLAB ^®^ code is attached for easy implementation.

## 1 INTRODUCTION

Quality Assurance of linear accelerators is an important part of the overall Radiotherapy Quality Management system 1, 2 and specific tests for linacs have been recommended in many international publications.^3^, ^4^ Measurement of the beam focal spot offset is not explicitly advocated, because its measurement is impractical and time‐consuming.^5^ However, beam focal spot position influences dosimetric and geometric properties of the beam (i.e., beam flatness and symmetry; radiation isocenter size and position). Ideally, it should be positioned at the collimator axis of rotation, so the size of the radiation isocenter is minimal and the propagation of the beam is along the collimator axis of rotation, as assumed and modeled by radiotherapy Treatment Planning Systems.

**Figure 1:**
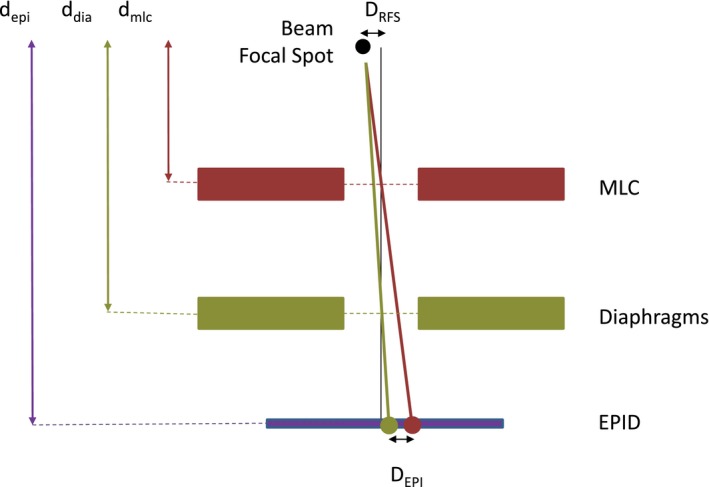
Diagram of the Elekta Agility^®^ head (schematic and not to scale) and illustration of beam focal spot position determination using the EPID. Vertical black line represents the collimator rotation axis. Red and green dots represent beam centers defined by MLC and diaphragms, respectively. Black dot represent the determined beam focal spot offset in relation to the collimator rotation axis.

A significant improvement of the beam focal spot offset measurement methodology proposed by Chojnowski et al.^6^ using an EPID‐based and phantom‐less technique, has since been shown to produce quick and accurate measurements. The idea was developed from the fact that if the radiation source is aligned with the collimator axis then the radiation isocenter position determined by the collimator rotation is independent of the type of field collimation used: jaws (diaphragms) or MLC. However, if the radiation source is misaligned with the collimator axis of rotation then the radiation isocenter position depends on the type of collimation, (see Fig. 1.) because the physical position and distance of jaws and MLC are different in relation to the radiation source.

The procedure of measuring the beam focal spot offset as described by Chojnowski et al.^6^ is specific to Varian machines. It uses four open fields; two of which use two sets of jaws (X and Y) to form the collimation aperture and the other two using the MLC. While an Elekta machine with an Agility^®^ head has only one set of diaphragms and MLC it is sufficient for determination of the beam focal spot offset when using a modified approach.

## 2 METHODS

### 2.A Linear accelerators

All tests were conducted on two linear accelerators at the Mid North Coast Cancer Institute at Coffs Harbour in NSW, Australia. The two Elekta Synergy^®^ models (Elekta Medical Systems, Crawley, UK) are equipped with the iView electronic portal imaging device (EPID). The iView EPID has a resolution of 1024 × 1024 pixels and the pixel size is 0.4 mm. 100 MU beams were delivered to an EPID using 6MV energy at a gantry angle 0^o^. The iView EPID is positioned at a fixed Source‐Imager‐Distance of 160 cm.

### 2.B Method

An Elekta machine with the Agility head has one set of diaphragms and so cannot form square field using diaphragms alone. However, one set of diaphragms can be used to determine the beam center in one direction, that is, inplane for collimator angle settings of 0^o^ and 180^o^ and cross‐plane for settings of 90^o^ and 270^o^. The beam center can then be built from this composite assessment.

To streamline the overall process, the same methodology as for diaphragms can be applied for the MLC. When two 100MU images are acquired at the opposite collimator angles of 0^o^ and 180^o^, the diaphragms determine the beam center in the inplane direction while the MLC determine the beam center in the cross‐plane direction. Utilizing two additional fields, with opposite collimator angles of 90^o^ and 270^o^, the diaphragms are then used to determine the beam center in the cross‐plane direction while the MLC determines the beam center in the inplane direction. By combining those two results, differences between beam centers defined by diaphragms and the MLC can be calculated and directly correlated with the beam focal spot position.

The calculation formula for the beam focal spot offset (eq. (1).) is the same as previously published by Chojnowski et al.^6^ but the schematic diagram (Fig. 1.) and proportionality factor “a” (eq. (2).) have been modified to reflect the geometry of the Elekta Agility^®^ head. The beam focal spot offset in any one direction is a product of the proportionality constant and the distance between beam centers, determined by the EPID:

(1) $$D_{\mathrm{BFSO}}=a*D_{\mathrm{EPI}}$$

where: D_BFSO_ = Beam focal spot offset D_EPI_ = Measured distance between beam centers using the EPID a = machine and procedure specific proportionality factor

(2) $$a=\frac{1}{\frac{\left(d_{\mathrm{epi}}-d_{\mathrm{dia}}\right)}{d_{\mathrm{dia}}}-\frac{\left(d_{\mathrm{epi}}-d_{\mathrm{mlc}}\right)}{d_{\mathrm{mlc}}}}$$

where: d_epi_ = distance from X‐ray target (beam focal spot) to the EPID d_dia_ = distance from X‐ray target (beam focal spot) to the diaphragms d_mlc_ = distance from X‐ray target (beam focal spot) to the MLC.

All acquired EPID images were analyzed by a custom MATLAB^®^ (The MathWorks, Inc., Natick, USA) script to determine the two beam centroids defined by diaphragms and the MLC, respectively. Only the central part of each image was analyzed. First, each image was filtered to remove noise using a two‐dimensional median filtering with a 3 × 3 size matrix. Each image was normalized, with the minimum pixel value being assigned the value 0 and the maximum pixel value being assigned the value 1. Each image was then resized, using bicubic interpolation, by a factor of 10 to increase the calculation resolution. Next, each image was made binary, with a threshold of 0.5 representing the Full Width Half Maximum of the radiation field. The center of each radiation field was then calculated as the centroid of the binary object (field) in both inplane and cross‐plane directions.

All four acquired images were visually inspected on the iView workstation for any signs of image artifacts that could potentially affect the result of the test. The filtering function in the code should help in removing single dead pixels, but otherwise all safeguards of detecting unsuitable images, that is, large panel shifts are removed from the code for simplicity.

The distance between the two beam centers defined by diaphragms and the MLC at the EPID level was then calculated as the difference between the two centers expressed in pixels multiplied by pixel size 0.4 mm and the resize factor 10.

To calculate beam focal spot offset (eq. (1)), the distance determined between the two centers was multiplied by the proportionality factor “a” (eq. (2)), which for the Elekta machine is equal to −0.9078 (eq. (2); d_mlc_ = 35.54 cm, d_dia_ = 47.05 cm and d_epi_ = 160 cm).

The Agility^®^ head has the MLC assembly closer to the radiation source compared to the diaphragms (in contrast to the Varian machines) therefore the proportionality factor is negative, which basically means that the beam focal spot position varies in the opposite way to the directional difference in radiation isocenters defined by diaphragms and the MLC.

A practical ready‐to‐go procedure and a MATLAB^®^ script for the Elekta Linac with the Agility^®^ head are attached in Appendices S1 and S2, respectively.

### 2.C Validation

The validation of the modified, phantomless method is based on previous work published by Nyiri^5^; being a quantified half‐beam block method. In this method a Farmer‐type ionization chamber is attached on a jig to the collimator and the difference in currents for two opposite collimator angles is recorded. The difference is proportional to the beam focal spot position in a particular direction. The proportionality factor is specific to the spatial sensitivity of the chamber, determined directly by diaphragms by a known value. In the validation work, diaphragms were used to determine the beam focal spot offset in both inplane and cross‐plane directions. To minimize the measurement uncertainty six consecutive measurements with the EPID were taken and also two measurements with the ionization chamber using two diaphragms. Measurements were performed on two Elekta Synergy^®^ linear accelerators with Agility^®^ head using two available energies, 6 and 15 MV.

## 3 RESULTS AND DISCUSSION

The mean difference of beam focal spot offset measured using EPID and ionization chamber was found to be 0.004 ± 0.052 mm (1 SD) (Table 1) and the beam focal spot offset reproducibility was on average ±0.045 mm (Table 2).

**Table 1:** Validation of the phantomless method of beam focal spot offset measurement with the ionization chamber method

**Measurement**	Linac 1 (6 MV)		Linac 1 (15 MV)		Linac 2 (6 MV)		Linac 2 (15 MV)	
	Cross‐plane	Inplane	Cross‐plane	Inplane	Cross‐plane	Inplane	Cross‐plane	Inplane
	(mm)	(mm)	(mm)	(mm)	(mm)	(mm)	(mm)	(mm)
Ion chamber	−0.071	0.264	−0.055	0.149	−0.024	−0.274	0.035	−0.250
EPID	0.039	0.229	−0.053	0.077	−0.021	−0.281	0.029	−0.274
Difference	−0.111	0.035	−0.002	0.072	−0.002	0.007	0.007	0.024
Ion chamber (1SD)	0.000	0.009	0.006	0.007	0.016	0.009	0.008	0.000
EPID (1SD)	0.085	0.068	0.029	0.016	0.060	0.025	0.059	0.019

**Table 2:** Reproducibility measurements of the 6 MV and 15 MV beam focal spots offset using the EPID on 2 different Elekta Synergy® linacs

**Measurement**	Linac 1 (6MV)		Linac 1 (15MV)		Linac 2 (6MV)		Linac 2 (15MV)	
	Crossplane (mm)	Inplane (mm)	Crossplane (mm)	Inplane (mm)	Crossplane (mm)	Inplane (mm)	Crossplane (mm)	Inplane (mm)
1	−0.048	0.320	−0.090	0.076	−0.070	−0.307	0.083	−0.256
2	0.140	0.168	−0.058	0.085	−0.079	−0.290	0.076	−0.308
3	0.038	0.185	−0.072	0.103	−0.079	−0.304	0.071	−0.279
4	0.143	0.313	−0.008	0.064	0.042	−0.277	−0.068	−0.276
5	−0.004	0.189	−0.029	0.059	0.025	−0.263	0.005	−0.273
6	−0.033	0.201	−0.062	0.078	0.032	−0.242	0.005	−0.255
Average	0.039	0.229	−0.053	0.077	−0.021	−0.281	0.029	−0.274
1SD	0.085	0.068	0.029	0.016	0.060	0.025	0.059	0.019

Measurements were performed using both diaphragms and MLC at the four cardinal collimator angles. The difference in position of the centroids for each field in cross‐plane and inplane directions can be used as a measure of the alignment of the beam focal spot. This is based on the principle that although the diaphragms and MLC share a common rotation axis their different distances from the effective radiation source position mean that their respective beam centers will project differently onto the EPID if the beam focal spot is misaligned with the collimator axis.

It is difficult to differentiate the accuracy of the method itself and the reproducibility of beam focal spot offset parameter. Some results were extremely accurate (i.e., Linac 1 15 MV cross‐plane and Linac 2 6 MV cross‐plane) and some less so (i.e., Linac 1 6 MV cross‐plane and 15 MV inplane). The same procedure and analysis methodology was followed up for both linacs for both energies. Therefore, it can be concluded that the method is more precise than the uncertainty of the beam focal spot position for any given linac and energy.

It was found that changing the resize factor parameter in the software from 2 to 20 shows no significant difference in result with a magnitude of difference of ±0.002 mm (1 SD).

It was noticed that the mean discrepancy in validating beam focal spot offsets against the ionization chamber method was four times higher on Elekta linacs with the Agility^®^ head (0.004 ± 0.052 mm) compared to published results for Varian linacs^6^ (0.001 ± 0.015 mm). No specific reason for this was found other than the fact that the reproducibility of beam focal spot position is substantially higher on the Elekta machines.

The procedure presented in Appendix S1 can be automated as a Stored Beam in “Service Mode” (ref. “Agility and Integrity R3.0 Instruction for Use – Service Mode”) to speed up delivery and acquisition of the four fields.

## 4 CONCLUSION

An innovative phantom‐less method of measuring beam focal spot offset using the EPID has been presented for Elekta linacs with the Agility^®^ head. It is a modification of the method described previously for Varian linacs, 6 which have two sets of jaws as opposed to one for the Agility^®^ head. It has the same advantages of being an accurate, practical and fast technique. It is recommended to include this test as part of the monthly linac QA.

## CONFLICT OF INTEREST

The authors declare no conflict of interest.

## Supporting information


**Appendix S1.** Beam focal spot offset procedure.

**Appendix S2.** Beam focal spot offset script.

## References

[1] Mayles W , Lake R , McKenzie A , et al. Physics Aspects of Quality Control in Radiotherapy, IPEM Report 81. IPEM: York, UK; 1999.

[2] Huq M , Fraass B , Dunscombe P , et al. Task Group 100 report: Application of risk analysis methods to radiation therapy quality management. Med. Phys. 2016;43(7):4209–4262. 27370140 10.1118/1.4947547 PMC4985013

[3] Klein E , Hanley J , Bayouth J , et al. Task Group 142 report: Quality assurance of medical accelerators. Med. Phys. 2009;36(9):4197–4212. 19810494 10.1118/1.3190392

[4] Smith K , Balter P , Duhon J , et al. AAPM Medical Physics Practice Guideline 8.a.: Linear accelerator performance tests. J Appl Clin Med Phys. 2017;18(4):23–39. 28548315 10.1002/acm2.12080 PMC5874895

[5] Nyiri B , Smale J , Gerig L . Two self‐referencing methods for the measurement of the beam spot position. Med. Phys. 2012;39(12):7635–7643. 23231311 10.1118/1.4766270

[6] Chojnowski J , Barnes M , Sykes J , Thwaites D . Beam focal spot position: The forgotten linac QA parameter. An EPID‐based phantomless method for routine Stereotactic linac QA. J Appl Clin Med Phys. 2017;18(5):178–183. 10.1002/acm2.12147 PMC5875833 28786168

