# Supplementary material for: Beam focal spot position determination for an Elekta linac with the Agility® head; practical guide with a ready‐to‐go procedure
Source: J Appl Clin Med Phys. 2018 May 14;19(4):44–7. doi: 10.1002/acm2.12344 (PMC6036348; doi:10.1002/acm2.12344)
Supplement: Supplementary file 1 — Appendix S1. Beam focal spot offset procedure. [file ACM2-19-44-s001.docx]

**Appendix 1**

- Rotate gantry to 0^o^ and extend the EPID iview panel
- Open standard 10 x 10 cm^2^ field in service mode and rotate collimator to -90^o^
- Acquire the portal image for 100MU, and repeat for collimator angles 0^o^, 90^o^ and 180^o^
- Send images in DICOM format to the MATLAB software and execute the code attached in appendix 2
