# Supplementary material for: Beam focal spot position determination for an Elekta linac with the Agility® head; practical guide with a ready‐to‐go procedure
Source: J Appl Clin Med Phys. 2018 May 14;19(4):44–7. doi: 10.1002/acm2.12344 (PMC6036348; doi:10.1002/acm2.12344)
Supplement: Supplementary file 2 — Appendix S2. Beam focal spot offset script. [file ACM2-19-44-s002.docx]

**Appendix 2**

function Radiation_Focal_Spot_Offset

clc;factor=10; % magnification factor

for i=1:4 % DICOM files must be named accordingly: RTIMAGE_1, RTIMAGE_2,….

image = double(dicomread(strcat('RTIMAGE_',num2str(i),'.dcm')));

% cutting the central part of the image to avoid peripheral artifacts

Rows=1024;Columns=1024;

image=image(100:Rows-100,100:Columns-100);

% reducing noise

image=filter2([1 1 1; 1 1 1; 1 1 1],image,'valid');

% normalizing image

max_image=max(max(image)); min_image=min(min(image));

image_norm=1-(image-min_image)/(max_image-min_image);

% changing resolution

image_norm=imresize(image_norm,factor);

% converting to binary image

image_bw=im2bw(image_norm,0.5);

% determining center of the field

reg_bw=regionprops(image_bw);

y(i)=reg_bw(1).Centroid(1); % crossplane direction

x(i)=reg_bw(1).Centroid(2); % inplane direction

end

% calculations

mean_x_MLC=(x(1)+x(3))/2; mean_y_MLC=(y(2)+y(4))/2;

mean_x_FS=(x(2)+x(4))/2; mean_y_FS=(y(1)+y(3))/2;

d_dia=43.2+7.7/2; % Diaphragm distance from the source

d_mlc=30.9+9.277/2; % MLC distance from the source

% 'a' is a proportionality factor to calculate focal spot offset

a=1/((160-d_dia)/d_dia-(160-d_mlc)/d_mlc);

% 'f' is a conversion factor from pixels to millimeters

f=0.4/factor; % resolution of the IVIEW panel is 0.4mm

D_EPI_Y= f*(mean_y_FS-mean_y_MLC); % Distance between field centers in crossplane direction

D_EPI_X= f*(mean_x_FS-mean_x_MLC); % Distance between field centers in inplane direction

D_RFS_Crossplane=a*D_EPI_Y % focal spot offset in crossplane direction

D_RFS_Inplane=a*D_EPI_X % focal spot offset in inplane direction
